# A Geometrical Divide of Data Particle in Gravitational Classification of Moons and Circles Data Sets

**DOI:** 10.3390/e22101088

**Published:** 2020-09-27

**Authors:** Łukasz Rybak, Janusz Dudczyk

**Affiliations:** Institute of Information Technology and Technical Sciences, Stefan Batory State University, 96-100 Skierniewice, Poland; lrybak@pusb.pl

**Keywords:** gravitational classification, classification, centroid-based classifier, data particle modelling, data particle divide

## Abstract

Thus far, the Universal Law of Gravitation has found application in many issues related to pattern classification. Its popularity results from its clear theoretical foundations and the competitive effectiveness of the classifiers based on it. Both Moons and Circles data sets constitute distinctive types of data sets that can be found in machine learning. Despite the fact that they have not been formally defined yet, on the basis of their visualization, they can be defined as sets in which the distribution of objects of individual classes creates shapes similar to circles or semicircles. This article makes an attempt to improve the gravitational classifier that creates a data particle based on the class. The aim was to compare the effectiveness of the developed Geometrical Divide method with the popular method of creating a class-based data particle, which is described by a compound of 1 ÷ 1 cardinality in the Moons and Circles data sets classification process. The research made use of eight artificially generated data sets, which contained classes that were explicitly separated from each other as well as data sets with objects of different classes that did overlap each other. Within the limits of the conducted experiments, the Geometrical Divide method was combined with several algorithms for determining the mass of a data particle. The research did also use the *k*-Fold Cross-Validation. The results clearly showed that the proposed method is an efficient approach in the Moons and Circles data sets classification process. The conclusion section of the article elaborates on the identified advantages and disadvantages of the method as well as the possibilities of further research and development.

## 1. Introduction

Thus far, there is no formal definition in the literature to describe Moons and Circles data sets. However, as stated in the original work about the library implementing methods that generate the aforementioned data sets, their hallmark is the fact that in the feature space in the ${\mathbb{R}}^{2}$ dimension, the distribution of objects described by different labels, belonging to individual classes, creates shapes similar to circles or semicircles, which is illustrated by the visualization of such a data set. Other characteristics of these data sets are the dearth of a linear decision boundary, the proximity of centroids of different classes to each other, and the proximity of objects of different classes in the vicinity of the centroids. By reason of the above-mentioned features, these data sets generate problems in the process of their classification with the application of centroid-based algorithms and linear classifiers [1].

One of many popular groups of centroid-based classifiers is the one applying the Universal Law of Gravitation approach [2]. Their growing popularity is due to their simplicity and high efficiency [3]. As a result, the gravity model finds application in both supervised [4,5], and unsupervised [6,7] machine learning techniques. As Gorecki and Luczak stated in the article devoted to the variant of gravitational classification [8], the gravitational data model was first proposed by Wright in 1977 [9]. The analysis of the currently published fieldworks allows supplementing the cited thesis that the complete explanation of the theory of Data Gravitation-based Classification (DGC)—the explanation of key definitions and lemmas—was made only in 2005 in the pages of [5]. The following key concepts were clarified at that time: data particle, data mass, data centroid, atomic data particle, data gravity, gravitational data field, and a number of lemmas for Data Gravitation-based Classification. Based on that, Peng et al. developed Data Gravitation-based Classification. In the same year, Wang and Chen [10], attempted to solve the problem of a significant decrease in the performance of the Nearest Neighbor (NN) method in data sets, in which the distribution of attribute values of objects of individual classes determines the overlapping of objects with different labels in the feature space, which in the case of particular data sets, may also determine the aforementioned problem of too near distribution of class centroids [1]. Then, a new method of gravitational classification, namely Simulated Gravitational Collapse (SGC), was developed.

Throughout the years, the DGC received a number of amendments and hands-on applications. In 2006, the practical application of the DGC in the Intrusion Detection System (IDS) prototype was demonstrated [11]. By adapting the classic DGC [5] to solve the problem of classification of unbalanced data sets, the following methods were developed: the Imbalanced DGC (IDGC) [12], Under-Sampling Imbalanced Data Gravitation Classification (UI-DGC) [13], and Synthetic Minority Over-sampling Technique methods integrated with DGC (SMOTE-DGC) [14]. Carrying on with work on the IDGC method, Peng et al. (2017) [15] implemented an algorithm with a view to identifying problems and threats in unbalanced network traffic data. In the literature, one can also observe several gravity classifiers implemented to the problem of small data sets classification, one of them being the Cognitive Gravitation Model (CGM) [16].

Thus far, three methods have been proposed in terms of creating a data particle. The first is a method that creates a data particle on the basis of a single element of the set, which is described by a compound of 1 ÷ 1 cardinality (1OT1P) [5].

The Maximum Distance Principium (MDP) algorithm creates a data particle based on the distance within the elements with the same label [5]. The approach creates a data particle on the basis of the object class with a 1 ÷ 1 compound (1CT1P), finding application, among others, in [17,18]. A number of approaches can be distinguished in the literature that focus on the determination of the centroid, e.g., [19,20], as mentioned in [17], and several approaches used to determine the mass of a data particle [17,18].

The popular Density-Based Spatial Clustering of Applications with Noise (DBSCAN) algorithm is the method worth recalling in considerations concerning the Moons and Circles data sets, although it does not belong to the gravitational approaches group. It is a clustering algorithm that is suitable for the spatial databases, in which the groups of the objects form the arbitrary shapes [21]. The mentioned approach has two main disadvantages. The first is the rapidly growing computational complexity—in the two-dimensional feature space, it is logarithmic, but in higher dimension and/or when the number of data set objects is large, the algorithm rapidly leads to quadratic complexity [22]. This article confirms that DBSCAN is a good approach to data sets whose objects create the arbitrary shapes, but on the condition that they are clearly separated from each other in the feature space, which is the second disadvantage of this algorithm.

Taking into account the fact that there is no linear decision boundary in Moons and Circles data sets and the fact that the gravitational classifiers connecting the data particle to the class do not allow for a sufficient curvature of the decision boundary, it can be hypothesized that the gravity model using the aforementioned method of creating a data particle may not be effective in the Moons and Circles data sets classification process. The above-mentioned fact became a direct inspiration for the authors of this article to take up the research problem, in which the authors were looking for an answer to the following question: Is it possible to effectively adapt gravity classifiers to the Moons and Circles data sets classification process in the feature space in the ${\mathbb{R}}^{2}$ dimension, using the Geometrical Divide method?

The purpose of the article was to compare the effectiveness of the developed Geometrical Divide (GD) method with the popular method of creating a class-based data particle, which was described by a compound of 1 ÷ 1 cardinality (1CT1P) in the Moons and Circles data sets classification process in the feature space in the ${\mathbb{R}}^{2}$ dimension.

As part of the research study, the authors conducted an experiment in which they compared the results obtained on the basis of the Geometrical Divide (GD) algorithm with the method that creates a data particle based on the class described by a compound of 1 ÷ 1 cardinality (1CT1P). They combined both methods with algorithms for determining the mass of a data particle, which were developed after the year 2017. The authors used the *k*-Fold Cross-Validation method and a number of classifier quality assessment measures.

The obtained results showed that centroid-based classifiers that apply the gravity model have the potential in the Moons and Circles data sets classification process described in the ${\mathbb{R}}^{2}$ dimension, where there is no linear decision boundary, the centers of gravity are located in close proximity to each other, and there are objects in their surroundings that belong to different classes.

## 2. Materials and Methods

The origins of the proposed Geometrical Divide method is based on the mathematical foundations of the Universal Law of Gravitation, which was developed by Isaac Newton in 1687 [2]; it says that between any pair of points with mass, on the line intersecting their centers, there is a force whose value is proportional to the product of their masses and inversely proportional to the square of the distance between their centers. In [5], adapting this theory to the area of machine learning, the principles of gravitational data classification, on which the proposed GD method is based, were defined.

### 2.1. Gravitation-Based Classification Principles

The data particle definition is the starting point for the Geometrical Divide method. In compliance with the methods developed thus far, a data particle may consist of a single element o or a set of elements $O=\left\{o_{1},\;\ldots ,\;o_{N}\right\}$ belonging to a certain, defined feature space S. Each element o is described by a feature vector $F=\left\{f_{1},\;\ldots ,\;f_{M}\right\}$ and label l. According to the theory idea of [5], the data particle P is the fundamental entity subject to analysis and ought to be considered as a defined type of data unit with three properties: data mass m, such that m∈ℝ\{0}, data centroid μ, and label l. Therefore, the data particle is expressed as a vector, as represented by the Formula (1):

(1) P={m, μ, l}.

Prediction of the label $l_{X}$ of the analyzed atomic data particle X, i.e., according to the definition of [5]—indivisible data element with mass $m_{X}=1$, is based on the determination of the value of the gravitational force of the data F between the data particle X and all data particles belonging to the examined feature space S. Thus, the following relationship (Equation (2)) is valid:

(2) $$\wedge_{P\in S}\vee_{F\in \mathbb{R}\backslash \left\{0\right\}}F\left(X,P_{i}\right)>0.$$

It needs to be noted that in the process of determining the force of gravity of data between data particles, unlike the classical theory of gravity, the gravity constant G is ignored [6]. As a result, the value of the gravitational force of the data between the atomic particle X and the i-th data particle $P_{i}$ is expressed by the following relationship, as shown in Equation (3):

(3) $$F\left(X,P_{i}\right)=\frac{m_{i}}{r^{2}},$$

where r is the distance between the atomic data particle X and the data particle $P_{i}$.

It is also possible to apply a different formula proposed in [17], which replaces the reciprocal of the square of the distance $\frac{1}{r^{2}}$ with the similarity function $Sim\left(X,P_{i}\right)$, according to this Equation (4):

(4) $$F\left(X,P_{i}\right)=m_{i}\cdot Sim\left(X,P_{i}\right).$$

Based on the data gravity force values determined in such way between the atomic data particle X and all N data particles belonging to the feature space S, the data particle $P_{i}$, for which the data gravity force was the highest, is selected [17], which is expressed by the following relationship, as shown in Equation (5):

(5) $$P_{y}=\underset{i\in \mathbb{N}\;\wedge \;i<N}{argmax}F\left(X,P_{i}\right).$$

In the last step, a decision is made to assign the data particle $P_{y}$ label to the classified atomic data particle X.

### 2.2. Data Particle Creation Based on Class

The approach to creating a data particle that has found application in the fieldworks is the one mentioned in the introduction, namely 1CT1P, which creates a data particle P on the basis of all objects o with the same label, belonging to the set O. The method assumes that in the input data set D, consisting of an N amount of objects o, there exist an M amount of sets O described with the label $l_{O}$, and the object o belongs to the set O only if the label of the object $l_{o}$ is identical with the label of the set $l_{O}$, which is expressed by the following relationship, Equation (6):

(6) $$o_{i}\in O_{j}\subseteq D\Leftrightarrow l_{o_{i}}=l_{O_{j}},$$

where i=1, 2, …, N and j=1, 2, …, M.

Then, for each set of objects O, a data particle P is created, which is expressed by the following relationship, Equation (7):

(7) $$\wedge_{O}\vee_{P}!P=\left[m,\;\sum_{o\in O}F_{o},l_{o}\right],$$

where $F_{o}$ is a feature vector describing the object o.

The presented method shares similarities with the MDP method [5]: in relation to the 1OT1P method, it reduces the number of data particles subject to further analysis. Therefore, it also curtails information about the relative position of objects described by the same label, which may have a negative bearing on the relevance of the data gravity computed in subsequent steps and ultimately on the accuracy of the entire classification process.

### 2.3. Determining Mass of Data Particle

Two algorithms for determining the mass of a data particle—Stochastic Learning Algorithm (GM SLA) and Batch-update Learning Algorithm (GM BLA) were proposed in 2017 [17]. It was then emphasized that the effectiveness of centroid-based algorithms depends on the characteristics of the data set.

Stochastic Learning Algorithm in the classifier learning phase, with each sample assigned incorrectly, modifies the mass values of two data particles $m_{i}$ and $m_{j}$ by the defined constant, ξ. The value of mass $m_{i}$ of a given data particle $P_{i}$, into which the sample X ought to be classified, is increased, which is expressed by the following relationship, as shown in Equation (8):

(8) $$m_{i}=m_{i}+\xi ,$$

while the value of the mass $m_{j}$ of the data particle $P_{j}$, to which the sample X has been assigned incorrectly, is decreased, which is described by the following Equation (9):

(9) $$m_{j}=m_{j}-\xi .$$

Batch-update Learning Algorithm modifies the mass value of individual classes once the classification run is complete, in the course of which it counts the number $\beta_{i1}$ of objects of the class $C_{i}$, which was classified to other classes, and also the number $\beta_{i2}$ of objects of the other classes erroneously classified to the class $C_{i}$. Then, based on the accumulated values and the constant ξ, it modifies the i-th mass of the data particle $m_{i}$ created on the basis of the class $C_{i}$, which is expressed by the following Formula (10):

(10) $$m_{i}=m_{i}+\left(\beta_{i1}-\beta_{i2}\right)\cdot \xi .$$

The authors of the presented algorithms indicated that the direction of further research may be to proofread the classifier, in which the mass of the data particle will depend on the numerical amount of the class. Therefore, in 2019, the effectiveness of the approach was put to the test, in which the mass value of a data particle results from the size of a particular class [18]. At that time, the aim of the research was to find out how the mass function influences the effectiveness of the Centroid-Based Classifier, which applies Newton’s Law of Universal Gravity [2]. The results of the research that was carried out on the *Iris* set showed that there is a potential for linear binding the mass of a data particle $m_{i}$ derived from the class of objects of its size $N_{i}$, which is expressed by the following relationship, as shown in Equation (11):

(11) $$m_{i}=N_{i}.$$

### 2.4. Geometrical Divide of Data Particle

The Geometrical Divide (GD) method presented by the authors of this article has its mathematical foundations in analytic geometry and assumes that each data particle P consists of at least 2 atomic data particles A, which is described by the feature vector $F=\left[f_{1},\;f_{2}\right]$, belonging to the feature space S in the ${\mathbb{R}}^{2}$ dimension. The process of splitting a single piece of data P is described below.

In the first step, the value of the depth level d, such that $d\in {\mathbb{N}}_{+}$, which satisfies the following equation, is defined in Equation (12):

(12) $${log}_{2}N_{min}<d,$$

where $N_{min}$ is the size of the smallest class C creating the data particle P.

Subsequently, for each existing data particle P based on the values of the atomic attributes of data particles $f_{1}$ and $f_{2}$ that assemble the data particle P, the data particle centroid μ is determined, which expresses the following relationship, as shown in Equation (13):

(13) $$\mu =\left[\sum_{i=1}^{\left|P\right|}A_{if_{1}}\cdot {\left|P\right|}^{-1},\sum_{i=1}^{\left|P\right|}A_{if_{2}}\cdot {\left|P\right|}^{-1}\right].$$

In the third step, based on the dispersion of the values of the atomic attributes of the data particles that assemble the data particle P, the geometric center C is determined for each of them, which is expressed by the following Formula (14):

(14) $$C=\left[f_{1max}-f_{1min},\;f_{2max}-f_{2min}\right],$$

where $f_{1max}$ is the maximum value of the feature $f_{1}$ of the atomic data particle A creating the particle P; $f_{1min}$ is the minimum value of the feature $f_{1}$ of the atomic data particle A creating the particle P; $f_{2max}$ is the maximum value of the feature $f_{2}$ of the atomic data particle A creating the particle P; and $f_{2min}$ is the minimum value of the feature $f_{2}$ of the atomic data particle A creating the particle P.

In the fourth step, the method determines the equation of the line constructed through the given points $\mu =\left[\mu_{1},\;\mu_{2}\right]$ and $C=\left[c_{1},\;c_{2}\right]$, which is expressed by the following relationship, as shown in Equation (15):

(15) $$\left(y-\mu_{2}\right)\left(c_{1}-\mu_{1}\right)-\left(c_{2}-\mu_{2}\right)\left(x-\mu_{1}\right)=0.$$

In the last, fifth step, the data particle is divided in regard to the defined straight line. Then, if the analyzed data particle A is above the constructed line, it will create a new data particle $P_{i}$, whereas if it is on the line or below it, it will create a data particle $P_{j}$. For this purpose, each atomic data particle $A=\left[a_{1},a_{2}\right]$, assembling a data particle P, is placed at the beginning of Formula (16):

(16) $$A\in \{\begin{array}{c} P_{i}\;if\;\left(a_{2}-\mu_{2}\right)\left(c_{1}-\mu_{1}\right)-\left(c_{2}-\mu_{2}\right)\left(a_{1}-\mu_{1}\right)>0\\ P_{j}\;if\;\left(a_{2}-\mu_{2}\right)\left(c_{1}-\mu_{1}\right)-\left(c_{2}-\mu_{2}\right)\left(a_{1}-\mu_{1}\right)\leq 0 \end{array}.$$

The pseudocode of the Geometrical Divide (GD) algorithm is illustrated in Algorithm 1:
**Algorithm 1** Geometrical Divide (GD)dataParticles: list of data particlesfeatureVectors: list of data particle’s feature vectors $F=\left[f_{1},\;f_{2}\right]$newDataParticles: list of data particles created by divide$N_{min}$: size of the smallest classd$:\;d\in {\mathbb{N}}_{+}$$\mathrm{AND}\;{log}_{2}N_{min}<d$
**FOR**i=2; i≤d  FOREACH P∈dataParticles
**DO**    
$\mu_{P}\;\leftarrow \;$
centroid(P)         
$//\;\mu \;=\left[\mu_{1},\;\mu_{2}\right]$
    
$C_{P}\;\leftarrow \;$
geometricCentroid(P)   $//\;C=\left[c_{1},\;c_{2}\right]$ dataParticle1 = List()           // create two empty lists dataParticle1      dataParticle2 = List()          // and dataParticle2 for new data particles    FOREACH F∈featureVectors
**DO**      $\mathrm{IF}\;\left(f_{2}-\mu_{2}\right)\left(c_{1}-\mu_{1}\right)-\left(c_{2}-\mu_{2}\right)\left(f_{1}-\mu_{1}\right)>0$
**THEN**        dataParticle1 ←
 add(returnParticle(F))      **ELSE**
dataParticle2 ←
 add(returnParticle(F))      **END IF**    **END FOREACH**    newDataParticles ←  add(dataParticle1) newDataParticles ←  add(dataParticle2)  **END FOREACH**  dataParticles ←  newDataParticles  newDataParticles ←  clear()**END FOR**

### 2.5. Computational Complexity of Geometrical Divide

The computational complexity of the proposed algorithm depends on the value of the depth level d and the size of the data set n. It is known that each element of the data set corresponds to exactly one atomic data particle, and each of them belongs to only one data particle. In reference to that, the deepest nested FOREACH loops, of which the outer will be executed i fold (i equals the number of existing data particles) and the inner will be executed j fold (j is the number of atomic data particles belonging to the data particle) will be executed n times in total—each data set object will be analyzed.

The analysis of the outer FOR loop shows that it will be executed d−1 fold, because at the level of the partition depth d=1, data particle division is not performed. Thus, the computational complexity of this algorithm is presented by Formula (17):

(17) $$Computational\;Complexity_{GD}=O\left(\left(d-1\right)n\right).$$

It should be emphasized that d≪n.

### 2.6. Data Sets

**Figure 1:**
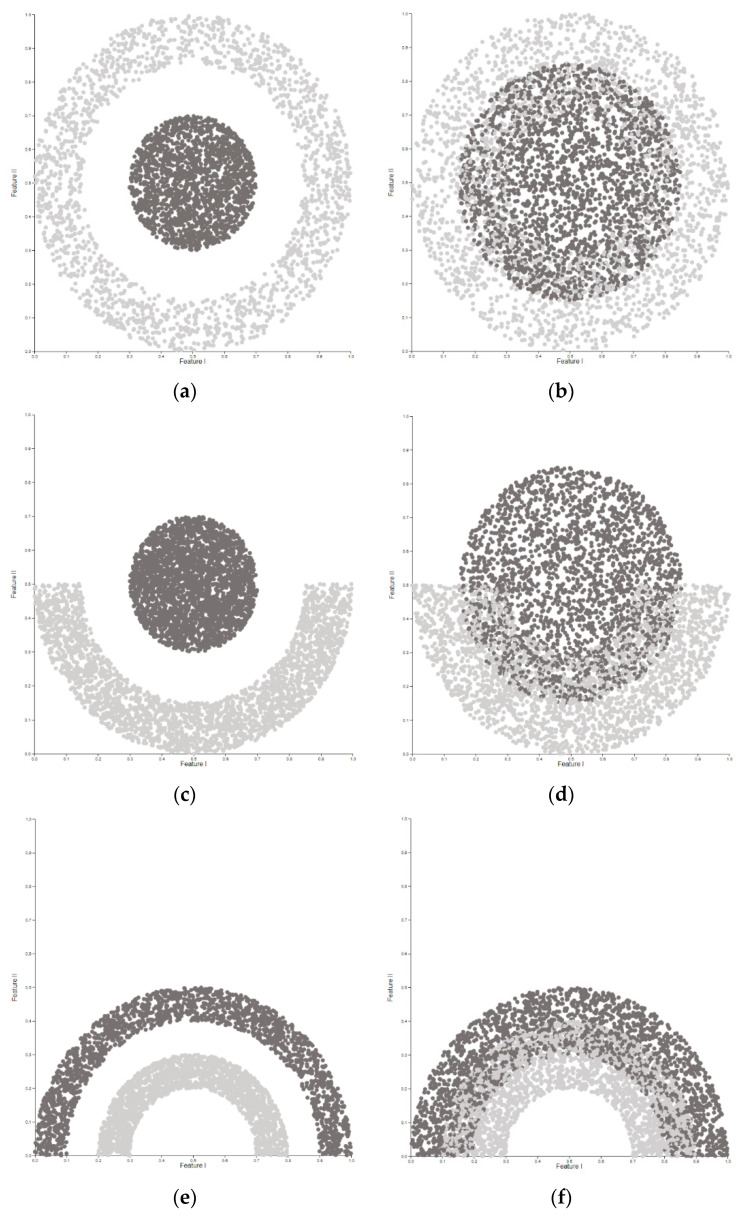

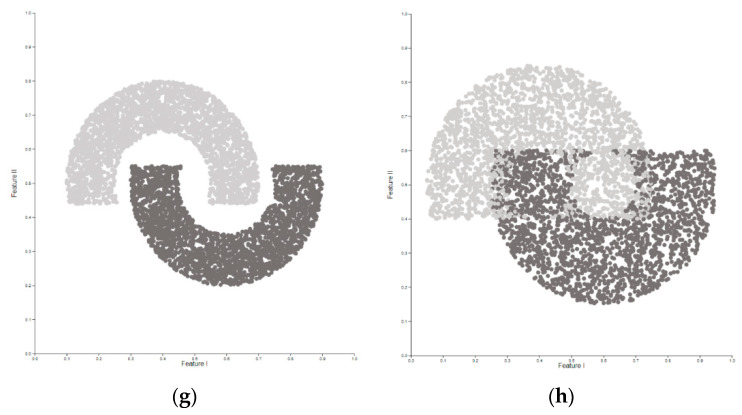
This figure presents the data sets that were used in the research: (**a**) circle_in_ring; (**b**) circle_in_ring_ns; (**c**) circle_and_moon; (**d**) circle_and_moon_ns; (**e**) two_moons; (**f**) two_moons_ns; (**g**) two_moons_mirrored; (**h**) two_moons_mirrored_ns.

In the conducted research, 8 artificially generated data sets were applied—4 in which the classes were explicitly separated from each other (data sets without ns (not separated) in their name) and 4 in which the objects of different classes did overlap each other (data sets with ns in their name). The distribution of feature values in the feature space in the ${\mathbb{R}}^{2}$ dimension was defined in a typical manner for Moons and Circles data sets. The sizes of the generated data sets were determined based on the characteristics of 42 real data sets used in previous publications in this field. The types of the generated data sets are presented in Figure 1a–h.

The characterization of the generated data sets is presented in Table 1.

In addition, the popular real data sets were processed as well. Two data sets from the repository of the University of California in Irvine (UCI) were chosen: The Banknote Authentication and The Breast Cancer Wisconsin [23]. In order for these data sets to meet the defined assumptions, their pre-processing was carried out. The preparation consisted in selecting two pairs of attributes from each data set that described them in the ${\mathbb{R}}^{2}$ feature space. In this way, 4 data sets were created. The first is banknote_authentication_12, which is based on the values of the variance and the skewness obtained as a result of the image wavelet transformation. The second data set is banknote_authentication_13, in which objects are described through variance and the kurtosis of the wavelet transformed image. The third is the breast_cancer_wisconsin_37, which described objects through the value of the cell size’s uniformity and the number of the bare nuclei. The last one is the breast_cancer_wisconsin_49, which is based on the value of the cell shape’s uniformity and the number of the normal nucleoli.

### 2.7. Evaluation of the Method

In the evaluation process of the proposed method, k-Fold Cross-Validation was used. It was also used to evaluate the models in many new publications [17,24]. The application of k-Fold Cross-Validation enables avoiding the model overfitting and improves the model’s generalization property [24]. The value of the parameter k was set at k=10. Such a parameterized method divided, in each iteration, the entire data set into two subsets: the test set, which constituted 10% of the entire base set, and the training set, consisting of the remaining 90% of the original set samples. In course of the first experiment, two data particle determination algorithms were compared: 1CT1P and GD.

The most popular and simplest metrics for pattern recognition system evaluation are accuracy (ACC) and error rate (ERR). It results from their key advantages—low complexity, the possibility of applying them in multi-class and multi-label classification processes, and easy-to-process scoring [25]. However, in the context of the Geometrical Divide evaluation in the Moons and Circles data sets classification process, in which the information about the prediction of individual classes is relevant, the dearth of informativeness of said measures in the subject issue, which excludes their use, needs be pointed out [25,26]. Therefore, said measures are not capable of providing relevant information that could enable an objective evaluation of the developed solution.

On account of the aforementioned downside, scientists apply complementary metrics in the field of machine learning, which was developed in 1955 and used to evaluate the information retrieval systems [27], including PRECISION, as shown in Equation (18):

(18) $$PRECISION=TP\cdot {\left(TP+FP\right)}^{-1},$$

where TP is true positive and FP is false positive, and RECALL, as shown in Equation (19):

(19) $$RECALL=TP\cdot {\left(TP+FN\right)}^{-1},$$

where FN is false negative.

In the process of model optimization, two objective functions may be taken into account, namely the maximization of the precision value and the maximization of the reference value. In practice, this task is difficult to implement, as most of the time, these functions are negatively correlated, which means that increasing the precision value often reduces the reference value and vice versa [28].

PRECISION and RECALL can be reduced to a single value $F_{\beta }-score$, which is the harmonic mean of the PRECISION and RECALL [29], as shown in Equation (20):

(20) $$F_{\beta }-score=\left(\beta^{2}+1\right)\cdot PRECISION\cdot RECALL\cdot {\left(\beta^{2}\cdot \left(PRECISION+RECALL\right)\right)}^{-1}.$$

## 3. Results

In the first stage, the parameter defining the depth of division in the proposed GD method was set at d=8. Each of the algorithms was combined with the Nearest Centroid (NC) method and three weight determination algorithms. The first one was the Stochastic Learning Algorithm (GM SLA), in which the parameter defining the maximum number of iterations in the learning process was defined as MaxIters=50, the mass value update coefficient was assigned the value ξ=0.0001, while the expected error threshold was set at ε=0.00. The second algorithm was the Batch-update Learning Algorithm (GM BLA), in which the data particle mass value update parameter was set at ξ=0.0001, while the last method of mass determination was the *n*-Mass Model (N-MM). The DBSCAN clustering algorithm with the minimum number of points required to create a dense region MinPts=10 and the parameter defining the maximum admissible distance between points within the same region Eps=0.150 was added to the comparison. The values of the $F_{1}-score$ obtained as a result of the first experiment for each individual method are presented in Table 2. The best result within each data set is highlighted in bold.

Having analyzed the acquired results, it can be concluded that the gravity classifier using the proposed GD approach obtained the best results on all artificially generated data sets. On data sets in which classes are separated—their objects do not overlap each other—the GD algorithm in all combinations and the DBSCAN algorithm obtained the measure result $F_{1}-score=1.000$, whereas for data sets in which the objects create the unseparated arbitrary shapes—sets with postfix ns—the best measure results of $F_{1}-score$ were returned by the GD approach in combination with the n-Mass Model (n-MM GD), which significantly outperforms the class-based data particle creation approach and the DBSCAN algorithm.

In the next step, the similar experiment was conducted on the real data sets whose objects do not create the shapes in the feature space. The parameters of the algorithms were set up identical to the first stage. Only the value of the d parameter was different, and in this case, d=3. The results of that part of the research are presented in Table 3. The best result within each data set is highlighted in bold.

Through the analysis of the results presented in Table 3, it can be observed that in the case of the tested real data sets, the $F_{1}-score$ results achieved by the algorithms using the proposed Geometrical Divide method outperform the results achieved by the algorithms that use the method of creating a class-based data particle by a compound of 1 ÷ 1 cardinality. The next conclusion based on an analysis of Table 3 is that the data sets, whose objects do not create the shapes in the feature space, are problematic for the DBSCAN algorithm.

The results presented in Table 2 and Table 3 were subjected to the Friedman Aligned Ranks statistical test [30] with the Holm post-hoc test [31] and with a statistical significance level of p=0.05. The test showed a significant improvement in the results obtained by the approaches using the proposed data particle divide algorithm in relation to the results obtained by the methods of creating a class-based data particle by a compound of 1 ÷ 1 cardinality and the popular DBSCAN algorithm. The same test showed that there is no statistically significant difference in the results obtained by the approaches belonging to the group of the methods that applied the Geometrical Divide algorithm (approaches with GD in the name).

**Figure 2:**
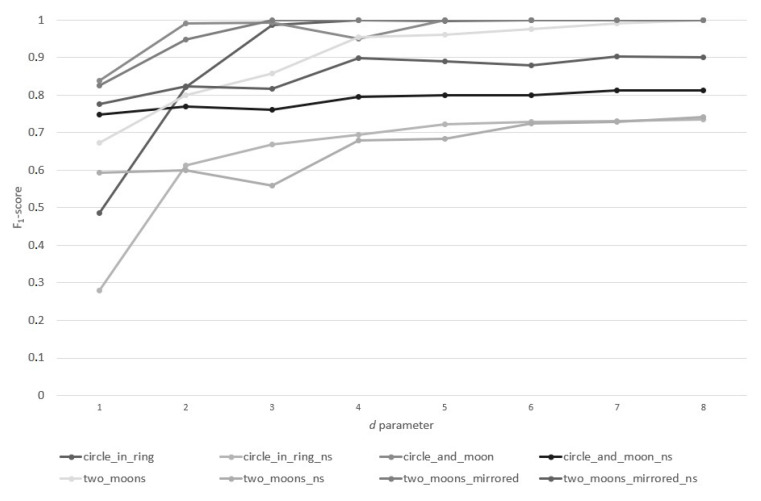
Change of the $F_{1}-score$ values for the subsequent values of the depth level d within individual data sets.

In the second stage of the research, the influence of the d parameter value on the $F_{1}-score$ obtained by the n-MM (GD) method was examined. On account of the characteristics of the examined data sets, the parameter d assumed integer values in the interval of [1 ÷ 8]. The results of this experiment are presented in Figure 2.

Based on the analysis of Figure 2, it can be noticed that the greatest improvement in the classifier’s results occurs already in the second iteration of the algorithm, i.e., in the first division of data particles. In each subsequent iteration, the improvement in results is relatively smaller. In some cases, noticeable decreases can be seen in the $F_{1}-score$. At the depth level of d=3, the said decrease can be observed for the two_moons_mirrored_ns and two_moons_ns data sets. At the depth level of d=4, similar behavior occurred for the circle_and_moon data set. Along with the subsequent levels of the depth d, the above-mentioned decreases are being gradually improved. In terms of all the examined data sets, at the depth level d=4, the $F_{1}-score$ value was higher than the value obtained before the splitting of the data particle.

**Figure 3:**
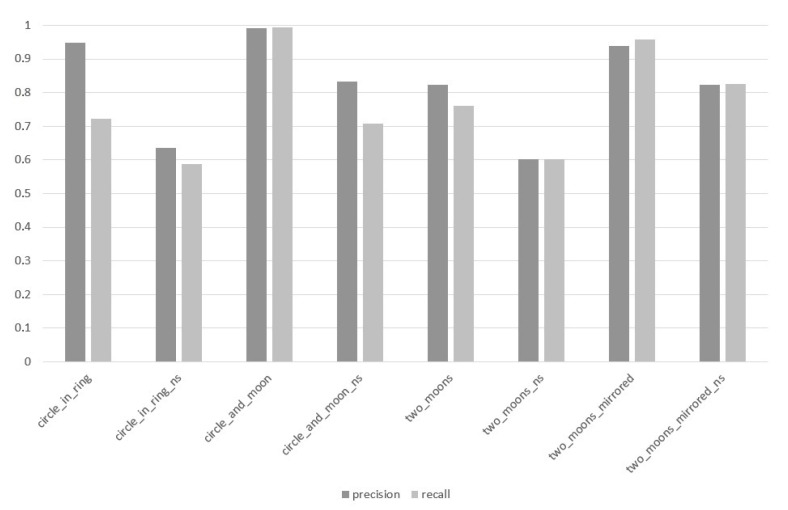
Values of PRECISION and RECALL measures for individual data sets, method: the *n*-Mass Model (*n*-MM) (Geometrical Divide, GD), parameter d=2.

**Figure 4:**
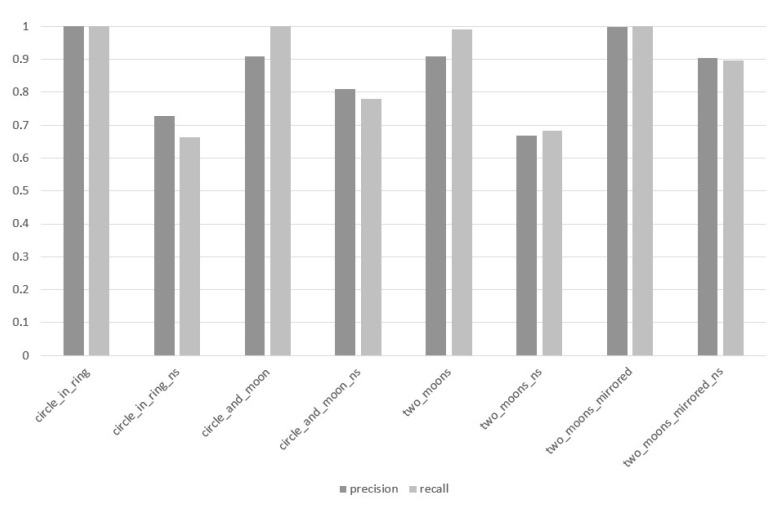
Values of PRECISION and RECALL measures for individual data sets, method: *n*-MM (GD), parameter d=4.

**Figure 5:**
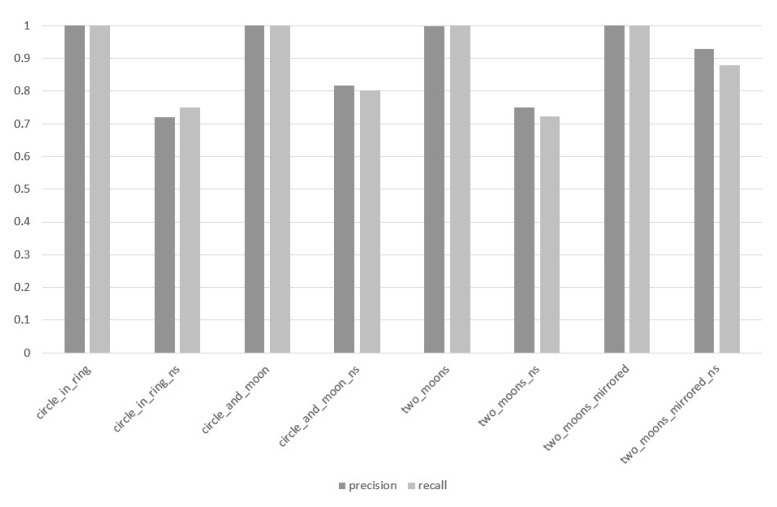
Values of PRECISION and RECALL measures for individual data sets, method: *n*-MM (GD), parameter d=8.

In the third stage of the experiment, the n-MM GD method was examined while paying special attention to the impact of the value of the depth parameter d on the value of the PRECISION and RECALL measures. The results of this stage of the research are visualized in Figure 3, Figure 4 and Figure 5.

For d=2 (Figure 3), the biggest difference in the results of both measures occurs in the case of the circle_in_ring data set and amounts to |PRECISION−RECALL|=0.226, whereas the mean difference between the above-mentioned measures within the entire experiment was 0.061.

At the depth level d=4 (Figure 4), in case of the circle_in_ring and two_moons_mirrored data sets, the significant difference between the PRECISION and RECALL values is not noticeable. For the circle_and_moon and two_moons data sets, the difference amounts to 0.090 and 0.082 respectively, giving the two highest difference scores between these measures within the experiment. In data sets where the classes are not separated from each other, the difference value between PRECISION and RECALL fluctuates in the interval of [0.009 ÷ 0.065]. On the other hand, the average difference value of said measures for the parameter d=4, in terms of all the analyzed data sets, amounted to 0.037. Thus, a balanced improvement of the classification can be noticed in the light of PRECISION and RECALL measures in contrast to the previous experiment, where the depth threshold d was set at d=2.

At the maximum depth level d=8 (Figure 5) that was examined, in case of data sets with clearly separated classes—circle_in_ring, circle_and_moon, two_moons and two_moons_mirrored—there is no significant difference between the obtained PRECISION and RECALL values. In terms of data sets in which the classes are not separated from each other (data sets with ns-postfix in its name), the differences in the values of both measures fluctuate in the interval of [0.015 ÷ 0.050], while the mean result of the difference between PRECISION and RECALL within the entire experiment with the parameter d=8 amounted to 0.015.

Based on the analysis of Figure 3, Figure 4 and Figure 5, it can be pointed out that the proposed method of splitting the GD data particle aims at a sustainable improvement of the results of the PRECISION and RECALL, which proves that the quality improvement is not based on the discrimination of any classes, and the classifier with similar effectiveness can predict all classes within a given data set. While analyzing Figure 2, Figure 3, Figure 4 and Figure 5, it can also be observed that with the increase of the depth of data particle division d, the difference between PRECISION and RECALL decreases, and the $F_{1}-score$ value increases concurrently.

## 4. Discussion

The article solves the problem of the low efficiency of classifiers based on the gravity model, which creates a class-based data particle described by a compound of 1 ÷ 1 cardinality. The disadvantage of such a method of binding is noticeable in the data sets classification process, in which the centers of gravity of classes with different labels are located in the proximity to each other, and there are objects belonging to other classes in their surrounding. The data sets possessing the above-mentioned features are Moons and Circles data sets.

The evaluation of the developed method was conducted with the use of approved classifier evaluation measures PRECISION, RECALL, and $F_{1}-score$. The obtained values of the above-mentioned measures showed an improvement in the results in contrast to the results obtained by applying the method of creating a class-based data particle with a compound of 1 ÷ 1 cardinality.

The first stage of the experiment demonstrated the main advantages of the Geometrical Divide algorithm. The first one is its resistance to a short distance between class centroids. In accordance to that, the GD method outperforms the data particle creation method based on class with a compound 1 ÷ 1 cardinality. The second advantage of the proposed algorithm is its resistance to the presence of objects of different classes in the vicinity of centroids. In this criterion, the GD algorithm significantly outperforms DBSCAN algorithm. It is also worth noting that in comparison to the Maximum Distance Principium method, the Geometrical Divide does not calculate the distance between atomic data particles and centroids in the phase of determining the data particles. In order to significantly improve the results of the classifier, the division of a data particle in the Geometrical Divide method does not always have to apply the maximum available depth level d.

The second stage of the experiment showed that for the value of the parameter d=4, which in terms of the analyzed data sets made up approximately the middle value of the interval of the available values, the results did not differ significantly from those obtained at the depth level d≥5. A noticeable improvement in the results can be observed already in the second iteration of the algorithm, during the first splitting of the data particle.

The third stage of the experiment displayed that the Geometrical Divide improves the gravity classifier in a balanced way; therefore, the improvement of its quality is not based on the discrimination of any of the classes, and the classifier is capable of predicting all classes within a given data set with similar effectiveness.

As far as further research is concerned, it is planned to apply the Geometrical Divide method in the process of classifying unbalanced data sets and/or higher dimension data sets. Another direction of analysis may be an attempt to develop a method of mass inheritance in the course of the splitting process. Furthermore, it is worth endeavoring to develop a method for selecting the parameter d based on the distribution of class attributes of the data set, which may contribute toward the further development of the presented method.

## Figures and Tables

**Table 1:** The results of the *F_1_-score* obtained by the algorithms applied on individual data sets.

Data Set	POSITIVES	NEGATIVES
circle_in_ring	1862	1864
circle_in_ring_ns	2388	2457
circle_and_moon	2584	2697
circle_and_moon_ns	2425	2500
two_moons	2886	1938
two_moons_ns	2384	2402
two_moons_mirrored	2512	2610
two_moons_mirrored_ns	2213	2252

**Table 2:** The results of the $F_{1}-score$ obtained by the algorithms applied on artificially generated data sets.

Data Set	DBSCAN	NC (1CT1P)	GM SLA (1CT1P)	GM BLA (1CT1P)	*n*-MM (1CT1P)	NC (GD)	GM SLA (GD)	GM BLA (GD)	*n*-MM (GD)
circle_in_ring	**1.000**	0.507	0.573	0.505	0.484	**1.000**	**1.000**	**1.000**	**1.000**
circle_in_ring_ns	0.509	0.497	0.485	0.498	0.314	0.724	0.725	0.724	**0.735**
circle_and_moon	**1.000**	0.851	0.674	0.876	0.837	**1.000**	**1.000**	**1.000**	**1.000**
circle_and_moon_ns	0.492	0.744	0.724	0.739	0.746	0.804	0.801	0.804	**0.806**
two_moons	**1.000**	0.686	0.523	0.705	0.673	**1.000**	**1.000**	**1.000**	**1.000**
two_moons_ns	0.496	0.601	0.604	0.588	0.594	0.734	0.736	0.734	**0.738**
two_moons_mirrored	**1.000**	0.824	0.822	0.826	0.824	**1.000**	**1.000**	**1.000**	**1.000**
two_moons_mirrored_ns	0.498	0.776	0.776	0.775	0.776	0.899	0.900	0.900	**0.907**

**Table 3:** The results of the $F_{1}-score$ obtained by the algorithms applied on real data sets.

Data Set	DBSCAN	NC (1CT1P)	GM SLA (1CT1P)	GM BLA (1CT1P)	*n*-MM (1CT1P)	NC (GD)	GM SLA (GD)	GM BLA (GD)	*n*-MM (GD)
banknote_authentication_12	0.372	0.864	0.862	0.864	0.866	0.904	**0.905**	0.904	0.897
banknote_authentication_13	0.303	0.810	0.810	0.810	0.800	0.830	0.831	0.829	**0.838**
breast_cancer_wisconsin_37	0.518	0.919	0.919	0.919	0.881	**0.937**	0.934	**0.937**	0.890
breast_cancer_wisconsin_49	0.469	0.871	0.871	0.871	0.816	**0.887**	0.886	0.886	0.872
